# The gut microbiome of farmed Arctic char (*Salvelinus alpinus*) is shaped by feeding stage and nutrient presence

**DOI:** 10.1093/femsmc/xtae011

**Published:** 2024-04-23

**Authors:** Stephen Knobloch, Sigurlaug Skirnisdóttir, Marianne Dubois, Lucie Mayolle, Laetitia Kolypczuk, Françoise Leroi, Alexandra Leeper, Delphine Passerini, Viggó Þ Marteinsson

**Affiliations:** Matís ohf., Microbiology Research Group, Vínlandsleið 12, 113 Reykjavík, Iceland; Department of Food Technology, University of Applied Sciences Fulda, Leipziger Strasse 123, 36037 Fulda, Germany; Matís ohf., Microbiology Research Group, Vínlandsleið 12, 113 Reykjavík, Iceland; ESBS/University of Strasbourg, 300 Bd Sébastien Brant, 67085 Strasbourg, France; University of Technology of Compiègne, Rue Roger Couttolenc, 60203 Compiègne, France; Ifremer, MASAE Microbiologie Aliment Santé Environnement, BP 21105, F-44000 Nantes, France; Ifremer, MASAE Microbiologie Aliment Santé Environnement, BP 21105, F-44000 Nantes, France; Matís ohf., Microbiology Research Group, Vínlandsleið 12, 113 Reykjavík, Iceland; Department of Animal and Aquaculture Sciences, Faculty of Biosciences, Norwegian University of Life Sciences, Arboretveien 6, 1430 Ås, Norway; Iceland Ocean Cluster, Department of Research and Innovation, Grandagarður 16, 101 Reykjavík, Iceland; Ifremer, MASAE Microbiologie Aliment Santé Environnement, BP 21105, F-44000 Nantes, France; Matís ohf., Microbiology Research Group, Vínlandsleið 12, 113 Reykjavík, Iceland; Faculty of Food Science and Nutrition, University of Iceland, Sæmundargata 2, 101 Reykjavik, Iceland

**Keywords:** aquaculture, gut microbiome, Arctic char, salmon, *Mycoplasma*, *Ruminococcaceae*, metagenomics

## Abstract

The gut microbiome plays an important role in maintaining health and productivity of farmed fish. However, the functional role of most gut microorganisms remains unknown. Identifying the stable members of the gut microbiota and understanding their functional roles could aid in the selection of positive traits or act as a proxy for fish health in aquaculture. Here, we analyse the gut microbial community of farmed juvenile Arctic char (*Salvelinus alpinus*) and reconstruct the metabolic potential of its main symbionts. The gut microbiota of Arctic char undergoes a succession in community composition during the first weeks post-hatch, with a decrease in Shannon diversity and the establishment of three dominant bacterial taxa. The genome of the most abundant bacterium, a *Mycoplasma* sp., shows adaptation to rapid growth in the nutrient-rich gut environment. The second most abundant taxon, a *Brevinema* sp., has versatile metabolic potential, including genes involved in host mucin degradation and utilization. However, during periods of absent gut content, a *Ruminococcaceae* bacterium becomes dominant, possibly outgrowing all other bacteria through the production of secondary metabolites involved in quorum sensing and cross-inhibition while benefiting the host through short-chain fatty acid production. Whereas *Mycoplasma* is often present as a symbiont in farmed salmonids, we show that the *Ruminococcaceae* species is also detected in wild Arctic char, suggesting a close evolutionary relationship between the host and this symbiotic bacterium.

## Introduction

Symbiotic microorganisms are essential for the health and well-being of all animals (Douglas 2018). In farm animals, microbiomes have been associated with different health characteristics which impact animal welfare and productivity (Jin Song et al. 2019, Chen et al. 2021, Wessels 2022). In aquaculture, fish are constantly exposed to high loads of microorganisms in the rearing water and need to maintain a healthy protective barrier to prevent infection and disease (Sundh et al. 2010, Langlois et al. 2021). Commensal microorganisms inhabiting fish mucosal surfaces, such as the skin, gills, and gut, can aid in this function by outcompeting opportunistic pathogens for nutrients or actively preventing their colonization through the production of antimicrobial compounds (Tarnecki et al. 2017, Perry et al. 2020). Apart from their role in disease prevention, the fish gut microbiome assists in nutrient uptake by breaking down complex carbohydrates and proteins in the gut, or through the production of vitamins and other essential nutrients (Clements et al. 2014, Yukgehnaish et al. 2020). This, in turn, leads to more efficient feed utilization and improved growth. Despite the importance of microbiomes in maintaining the health and productivity of farmed fish, knowledge of their community composition and function across the diverse range of host species currently being farmed remains limited.

Within the group of salmonids, which includes several high-value aquaculture species, the gut microbiome of Atlantic salmon (*Salmo salar*) has so far received most attention (Rudi et al. 2018, Dvergedal et al. 2020). Previous studies have shown that the Atlantic salmon gut contains few autochthonous, or resident, gut microorganisms (Gajardo et al. 2016, Karlsen et al. 2022), with *Mycoplasma* being one of only few recurring gut commensal (Llewellyn et al. 2015, Dehler et al. 2017, Fogarty et al. 2019, Rasmussen et al. 2021). Compared to Atlantic salmon, the microbiome of Arctic char (*Salvelinus alpinus*), a cold-water fish species, is less well-studied. First studies, using electron microscopy and a cultivation-based approach, demonstrated substantial numbers of bacteria inhabiting the gut of Arctic char (Ringø et al. 2001, 2006). Research on wild Arctic char using high-throughput sequencing technology has shown a heterogeneity of the gut microbial diversity across geography, season and habitat (Hamilton et al. 2019, Element et al. 2020, 2021). Similar to Atlantic salmon, *Mycoplasma* was also among the dominant gut symbionts. In an aquaculture environment, the gut microbiota of Arctic char has been studied to determine the impact of feeds and probiotics on the microbial community (Nyman et al. 2017, Knobloch et al. 2022). However, it is not yet known which bacteria constitute the resident gut microbiota in farmed Arctic char or what role they might play in maintaining health, well-being and productivity of the fish. Understanding these interactions, particularly during the early life stages, could be valuable for characterizing a healthy Arctic char gut microbiome, formulating precision diets, or modulating less productive gut microbiota through the selection and transplantation of probiotic strains.

The objective of this study was to describe the microbiome of farmed juvenile Arctic char, identifying the stable members of the resident gut microbiota and analysing their putative role in the fish gut microbiome through 16S rRNA gene amplicon sequencing, fluorescence *in situ* hybridization (FISH) and metagenomic analysis.

## Material and methods

### Sample collection

Juvenile Arctic char (*S. alpinus*) were collected from an ongoing feeding trial (Knobloch et al. 2022) at 104 days posthatch (dph) (T0, *N* = 22), 132 dph (T1, *N* = 15), and 157 dph (T2, *N* = 15). Before the beginning of the feeding trial (T0), one fish was collected from each of the 22 experimental tanks. At T1 and T2, five fish were collected from each of the three replicate control tanks. All fish had received the same control diet, consisting of fish meal, fish oil, gelatinized wheat, minerals, and vitamins as described in Knobloch et al. (2022), and were reared under identical conditions with a continuous freshwater exchange and a water temperature of 8.6 ± 0.5°C. The fish were fasted 12 h prior to weighing and sample collection. Fish were then euthanized with 500 ppm of phenoxyethanol and transported to the laboratory in sterile plastic bags on ice. Skin samples were collected by scraping along the lateral line using a sterile scalpel. The fish were then rinsed with 70% ethanol followed by sterile laboratory grade water to remove loosely attached bacteria before dissecting and removing the mid- and hind-gut section. For histology, ∼5 mm sections were removed from the hind-gut and fixed for 24 h in freshly prepared 4% paraformaldehyde solution in 1x PBS at 4°C before being transferred to 70% ethanol for long-term storage. At time T0, T1, and T2, 1 l of tank water was collected and filtered on 0.2 µm cellulose filters (Advantec). In total, 11 feed samples were collected in sterile containers over the study period. Skin, gut, water filter, and feed samples were frozen at −80°C until DNA extraction and sequencing. The experiment was performed according to European and Icelandic guidelines under the licence UST201707 from the Icelandic Environment Agency and FE-1134 from the Icelandic Food and Veterinarian Authority.

To compare the gut microbial community in relation to gut content 24 additional fish from time point T2, which had been collected and frozen at −80°C at the end of the experiment, were dissected as mentioned above. The state of digestive content was described as “full” if digesta was present along the mid- and hind-gut section, as “partially full” if digesta was present in parts of the mid- or hind-gut section, and as “empty” if no digesta was observable.

To compare the farmed Arctic char gut microbiota to those of wild specimens, 35 additional fish, ranging in weight from 1.8 to 15.5 g and collected from a fresh water spring in the south of Iceland (Kreiling et al. 2021), were dissected and processed as mentioned above.

### DNA extraction, PCR, and sequencing

DNA from the whole mid- and hind-gut with digesta, if present, was extracted as previously described in Leeper et al. (2022). In brief, defrosted guts were homogenized by bead-beating and then processed with the QIAamp PowerFecal Pro DNA Kit (Qiagen). Two negative extraction controls were run alongside these samples. DNA from skin, water filter, and feed samples were extracted using the MasterPure Complete DNA & RNA Purification Kit (Epicentre) following the manufacturer’s instructions for DNA extraction. PCR was performed on all samples as described in Knobloch et al. (2021) using the universal prokaryotic primer pair S-D-Bact-0341-b-S-17 (5′-CCTACGGGNGGCWGCAG-3′) and S-D-Bact-0785-a-A-21 (5′-GACTACHVGGGTATCTAATCC-3′) (Klindworth et al. 2013) and high-fidelity Q5 polymerase (New England Biolabs). All samples, including the negative PCR products of the extraction controls, were barcoded with Nextera XT v2 indices (Illumina), normalized using Sequel-Prep Normalisation Plates (Thermo Fisher Scientific), and sequenced on a MiSeq desktop sequencer (Illumina) with v3 chemistry to generate 300 bp long paired-end reads.

DNA of three selected samples with a high relative abundance of the three dominant gut symbionts were subjected to shotgun metagenomic sequencing. In short, bacterial DNA was enriched using the NEBNext Microbiome DNA Enrichment Kit (New England Biolabs) followed by library preparation using the Nextera Flex kit (Illumina) according to the manufacturer’s instructions. Libraries were pooled and sequenced on a MiSeq sequencer as mentioned above. Sequencing generated 2.88 Gbp raw data across 9 781 473 paired-end reads.

### Inference of 16S rRNA ASVs and microbial community analysis

Raw 16S rRNA reads were filtered, trimmed, and processed into amplicon sequence variants (ASVs) with DADA2 v. 1.12.1 (Callahan et al. 2016) implemented in R (R Core Team 2020). In short, primer sequences were removed and the forward and reverse reads trimmed after 260 bp and 240 bp, respectively. The settings maxEE and truncQ were both set to 2. After learning and filtering errors with default settings, forward and reverse reads were merged, sequences outside of the target amplicon range removed and chimeras detected with the function *removeBimeraDenovo*. Taxonomic assignment was performed with the function *assignTaxonomy* against a training set of the Silva SSU database version 138 (Quast et al. 2013). ASVs assigned to the kingdom *Eukaryota*, order *Chloroplast* or family *Mitochondria*, as well as 31 ASVs detected predominantly in the negative control samples, were removed. Microbial community analysis, including statistical analysis and plotting community composition, alpha diversity and beta diversity, was performed with R packages phyloseq version 1.42.0 (McMurdie and Holmes 2013) and vegan version 2.6–4 (Oksanen et al. 2013). Pairwise multilevel comparison was conducted with the R package pairwiseAdonis version 0.4.1 (https://github.com/pmartinezarbizu/pairwiseAdonis). A phylogenetic tree of all ASVs for calculating weighted UniFrac distances was created with DECIPHER (Wright 2016) and FastTree 2 (Price et al. 2010). Differential abundance analysis was performed with DeSeq2 version 1.38.1 (Love et al. 2014). The Venn diagram was produced with the R package ampvis2 version 2.7.35 (Andersen et al. 2018) with an abundance cutoff of 0.11% and a frequency cutoff of 1%.

### MAG binning, functional genome analysis, and phylogenetics

To construct the draft genomes of the three dominant fish gut symbionts, metagenomic raw reads were quality filtered with Trimmomatic (Bolger et al. 2014) with settings LEADING:3 TRAILING:3 SLIDINGWINDOW:4:15 MINLEN:100, leading to the removal of 17.59% of the raw data, and coassembled using Megahit version 1.1.3 (Li et al. 2015). Quality filtered reads of each sample were then mapped back to the coassembled contigs using Bowtie 2 (Langmead and Salzberg 2012). Binning of metagenome-assembled genomes (MAGs) was performed in Anvi’o version 7 (Eren et al. 2015) following the “Anvi’o User Tutorial for Metagenomic Workflow” (https://merenlab.org/2016/06/22/anvio-tutorial-v2/). In brief, k-mer frequencies were calculated and open reading frames (ORFs) identified in the contigs using the anvi-gen-contigs-database command. HMM (hidden Markov model) profiles were generated using the command anvi-run-hmms and genes annotated with the command anvi-run-ncbi-cogs. Taxonomic annotation was performed with centrifuge (Kim et al. 2016). Anvi’o profiles were merged and imported into the anvi’o interactive interface. MAGs were then manually binned based on tetranucleotide frequency, taxonomic assignment, and coverage. Genome completeness and contamination of the three resulting MAGs were calculated with CheckM version 1.1.0 (Parks et al. 2015) using a lineage-specific workflow. Average genome coverage for each MAG was calculated, by mapping the quality filtered reads to the MAG using Bowtie 2 and calculating coverage with the samtools depth function (Li et al. 2009). Closest cultivated relatives based on near full-length 16S rRNA genes of each MAG were determined with EzBioCLoud (Yoon et al. 2017). Average amino acid identity (AAI) was calculated using the tool Genome Matrix (Rodriguez-R and Konstantinidis 2016) on the web server http://enve-omics.ce.gatech.edu/g-matrix. Closely related genomes for comparison were chosen based on 16S rRNA gene similarity, as mentioned above. ORFs for each MAG were called with prodigal version 2.6.3 (Hyatt et al. 2010). Genome statistics were determined with QUAST (Gurevich et al. 2013) and RAST (Aziz et al. 2008). Clusters of orthologous groups of proteins (COGs) were predicted using RPS-BLAST+ against the 2014 release of the COG database (Galperin et al. 2015). COG category and functional descriptions were inferred using the cdd2cog.pl script (Leimbach 2016). Assigning KEGG Orthology (KOs) was performed using the BlastKOALA web service (Kanehisa and Goto 2000, Kanehisa et al. 2016). For those genes not found in specific KEGG pathways, a tBLASTn search of the corresponding candidate KO proteins against the MAGs was performed to verify their absence. Secondary metabolite gene clusters were searched with the web application of antiSMASH version 7 (Blin et al. 2017) with default settings. Genes involved in short chain fatty acid (SCFA) production were searched through the gutSMASH web server (Pascal Andreu et al. 2021). Carbohydrate-active enzymes were predicted with the dbCAN3 web server (Zheng et al. 2023) with all detection tools selected.

Phylogenetic trees based on the 16S rRNA gene sequence of each MAG was constructed in ARB (Ludwig et al. 2004). In brief, sequences were aligned against the global SILVA SSU alignment with the SINA web-tool (Pruesse et al. 2012) and merged with the SILVA SSU database version 138.1 (Quast et al. 2013) in ARB. Maximum-likelihood trees were calculated with PhyML (Guindon and Gascuel 2003) for each MAG including closely related sequence.

### Histology and FISH

Fixed gut sections were dehydrated in successive baths of ethanol and xylene, and then embedded in paraffin. Sections of 5 µm thickness were cut on a CM1800 microtome (Leica) with MX35 Ultra microtome blades (Thermo Scientific), followed by deparaffinizing in xylene and a washing step in 100% ethanol. FISH of probes to the bacterial 16S rRNA subunit was conducted as previously described in Knobloch et al. (2019) with Cy3-labelled probes targeting the *Mycoplasma* sp. (equal mixture of probes MYC542 and MYC629), the unclassified *Ruminococcaceae* (probe RUM1447) and the *Brevinema* sp. (probe BRV1455), as well as the previously described Alexa488-labelled universal bacterial probe EUB338 (Amann et al. 1990) (Table S3, Supporting Information). The hybridization buffer contained 40% formamide. Hybridized sections were stained with Fluoroshield antifade containing DAPI (Sigma) and visualized with a model BX51 epifluorescence microscope (Olympus). Epifluorescence images were processed with daime v. 2.2 (Daims et al. 2006).

## Results

### Temporal succession of the gut microbiota and dominance of three bacterial taxa

**Figure 1.**
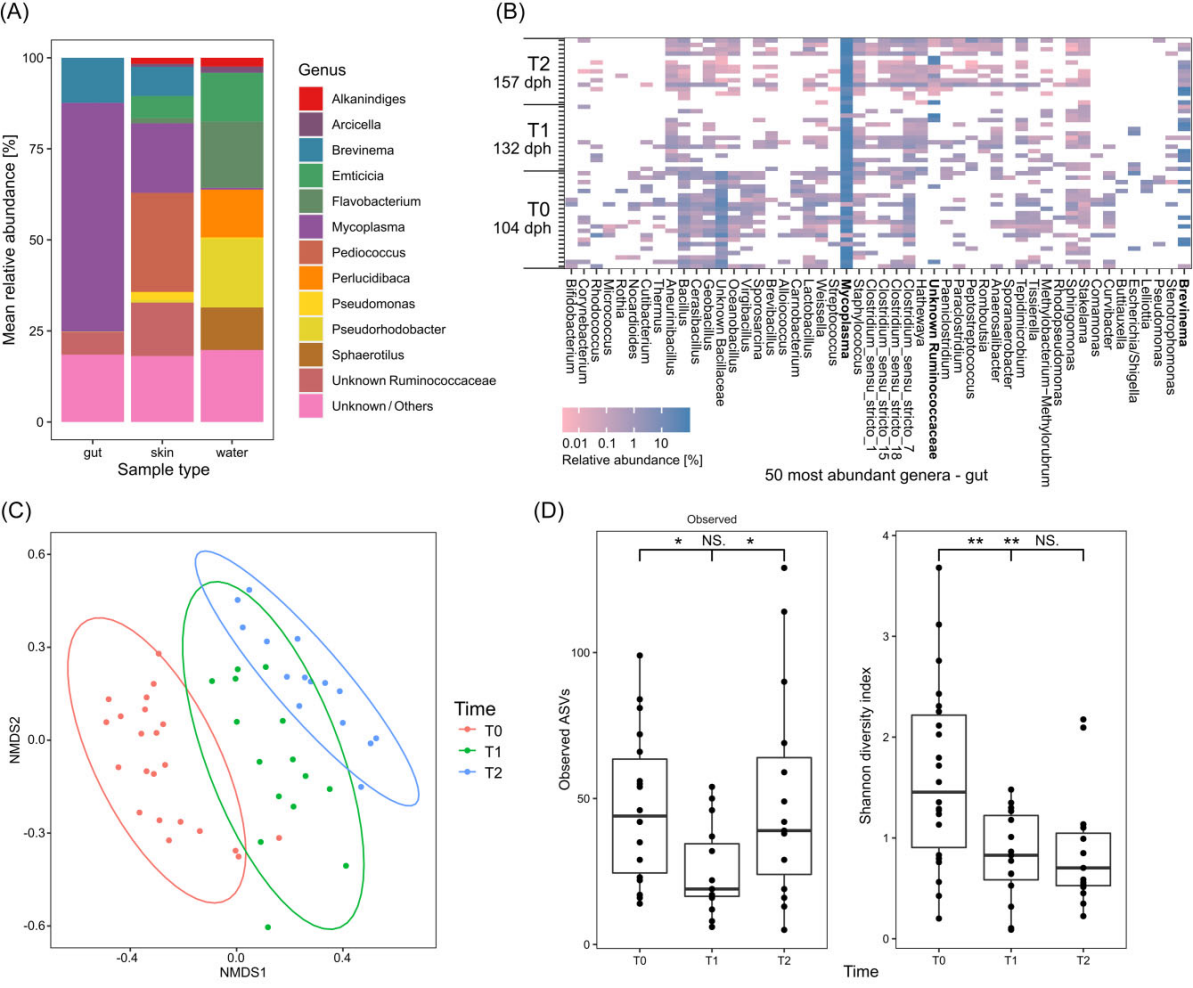
Microbial community composition and diversity of juvenile Arctic char. (A) Mean relative abundance of the 12 most abundant genera detected in gut, skin, and tank water samples across all time points; unclassified and low abundant taxa are summarized as “Unknown/Others” (gut samples: *n* = 52, skin: *n* = 18, and water: *n* = 3). (B) Relative abundance (log scale) of the 50 most abundant genera across all samples at time T0 (104 dph), T1 (132 dph), and T2 (157 dph). (C) Nonmetric multidimensional scaling (NMDS) plot of Bray–Curtis dissimilarities between gut microbial communities (stress = 0.2). (D) Alpha diversity metrices of gut microbial communities between times points; significance based on one-way ANOVA and Tukey’s *post hoc* test (*: *p*_adj_ < 0.05; **: *p*_adj_ < 0.01; NS: nonsignificant).

To describe the gut microbial community composition of juvenile farmed Arctic char, 16S rRNA gene sequence amplicons were analysed at three different time points over the course of 8 weeks from 52 farmed fish with an average weight of 2.2 ± 0.2 g at time T0, 4.5 ± 1.1 g at time T1, and 9.8 ± 0.7 g at time T2. The gut microbiome was dominated by the genus *Mycoplasma*, comprising 62.8% of the average relative abundance across all age groups (Fig. 1A). Within *Mycoplasma*, a single ASV made up 97.1% of the relative abundance, with most other ASVs having only a single nucleotide difference (data not shown). Though not detected in all samples, the second and third most abundant taxa were *Brevinema* and an unclassified *Ruminococcaceae*, both also dominated by a single ASV, and accounting for 12.3% and 6.3% of the average relative abundance, respectively. All other genera made up less than 20% of the average relative abundance.

There was considerable variability in the microbial community composition both between age groups and between individuals, with the *Mycoplasma*-associated ASV being the only ASV shared between all samples (Fig. 1B). The presence of the *Brevinema* sp. and unclassified *Ruminococcaceae* appeared to increase over time, with a higher percentage of fish containing either one of the species at T2 (87%) than at T0 (41%) (Fig. 1B). Other taxa, such as an unknown *Bacillaceae* and *Bacillus* sp. decreased markedly over time. A significant difference in the microbial communities between age groups was detected based on Bray–Curtis dissimilarities (PERMANOVA, F(2, 49) = [3.4148], *P* = .001) (Fig. 1C) and weighted UniFrac distances (PERMANOVA, F(2, 49) = [2.1006], *P* = .047) (Fig. S1, Supporting Information). The number of observed ASVs ranged from 5 to 129 with an average of 42 ASVs across all age groups. There were significant differences between the number of observed ASVs (ANOVA, F(2, 49) = [4.189], *P* = .0209) and Shannon diversity (ANOVA, F(2, 49) = [7.534], *P* = .0014) between time points, with the Shannon diversity significantly decreasing from T0 to T1 and to T2 (Fig. 1D).

Interestingly, *Mycoplasma, Brevinema*, and the unclassified *Ruminococcaceae* were also present on the skin of the fish, together accounting for 41.8% of the relative abundance, whereas they were only detected at a low relative abundance of 0.7% in the tank water samples (Fig. 1A).

### Absence of gut content strongly influences the gut microbial composition

**Figure 2.**
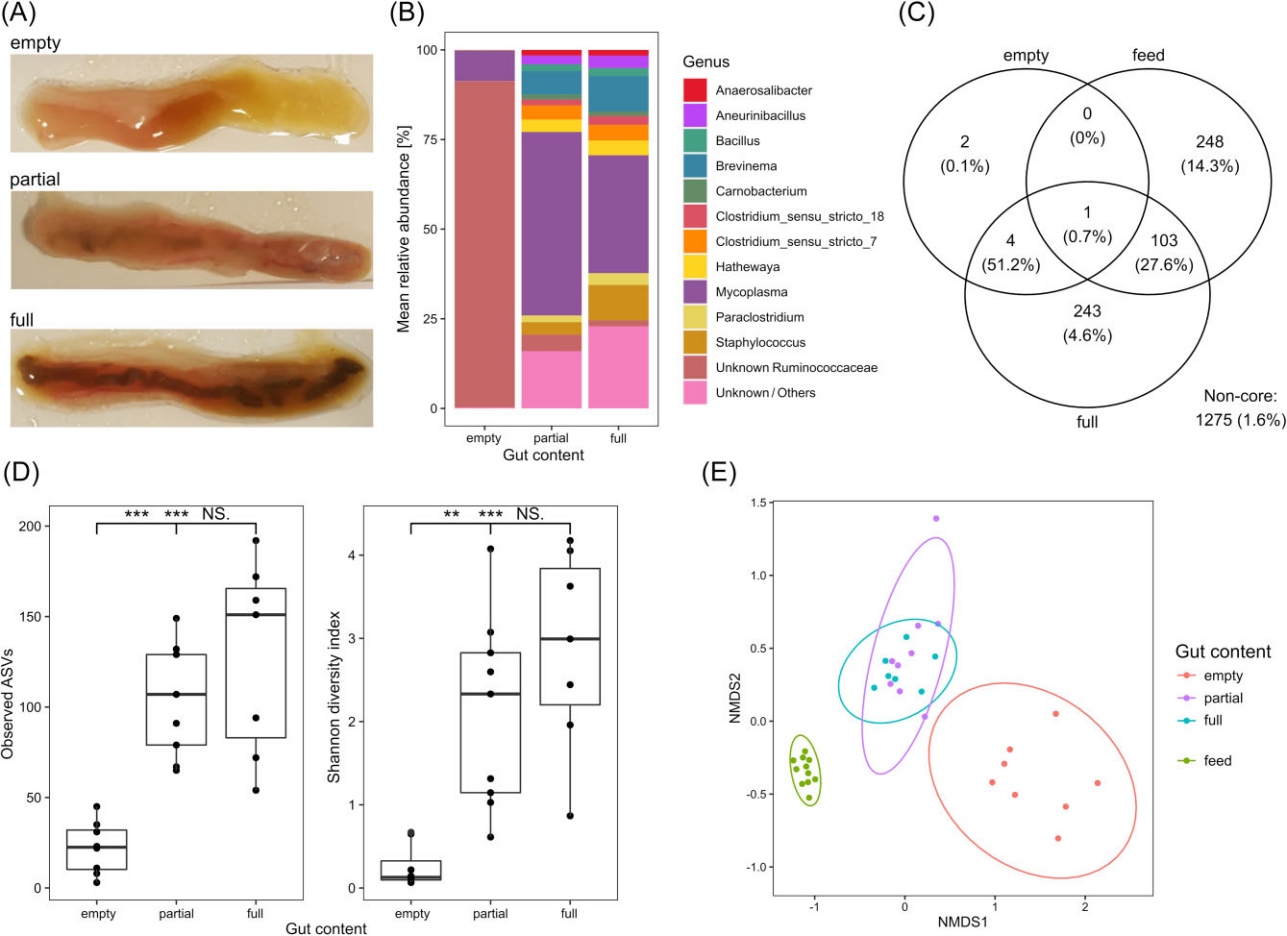
Microbial community composition of Arctic char guts with different amounts of gut content. (A) Example of gut sample characterized as empty, partially filled, and full. (B) Mean relative abundance of genera detected in gut samples characterized as empty (*n* = 8), partially filled (*n* = 9), and full (*n* = 7) at time T2; unclassified and low abundant taxa are summarized under “Unknown/Others”. (C) Venn diagram of shared taxa between empty guts, full guts, and feed (partially filled gut group excluded); values indicate the number of shared and not shared ASVs; percentages in brackets indicate percentage of relative abundance. (D) Alpha diversity metrices of gut microbial communities between empty, partially filled, and full guts; significance based on one-way ANOVA and Tukey’s *post hoc* test (**: *p*_adj_ < 0.01; ***: *p*_adj_ < 0.001; NS: nonsignificant). (E) NMDS plot of Bray–Curtis dissimilarities between microbial communities in empty, partially filled, and full guts, as well as feed samples (stress = 0.08).

Due to the large observed interindividual variability in gut microbial composition, the microbial community was examined in relationship to gut content filling on 24 additional fish from time point T2 of which eight fish were characterized as having empty mid- and hind-guts, nine as having partially filled guts and seven as having full mid- and hind-guts (Fig. 2A). The gut microbial community of fish with empty guts was dominated by the unclassified *Ruminococcaceae*, which was significantly more abundant in these samples than in samples of fish with partially full or full guts (DESeq2, *p*_adj_ < 0.001) (Fig. 2B). In addition, the number of observed ASVs (ANOVA, F(2, 21) = [19.11], *P* < .0001) and Shannon diversity (ANOVA, F(2, 21) = [14.84], *P* < .0001) was significantly different between the state of gut filling, with fish with empty guts having significantly lower values compared to fish with partially full or full guts (Fig. 2D). This discrepancy was also highlighted by a significant difference in the microbial community composition within the gut content and feed groups based on Bray–Curtis dissimilarities (PERMANOVA, F(3, 31) = [26.736], *P* = .001), with pairwise comparisons showing significant differences between empty and both full and partially filled guts (*p*_adj_ = 0.006 each) and between the feed samples and all three gut groups (*p*_adj_ = 0.006 each), but not between the full and partially filled guts (*p*_adj_ = 1) (Fig. 2E).

To evaluate the contribution of microorganisms in the fish feed to the gut microbial community, empty guts, full guts, and feed samples were compared to each other (Fig. 2C). This showed that only one ASV, a member of the *Clostridiaceae*, was detected in all sample types above a relative abundance of 0.1%. The three dominant ASVs in the gut, *Mycoplasma, Brevinema*, and the unclassified *Ruminococcaceae*, as well as a member of the genus *Rhodococcus* were shared between the empty and full guts and contributed to over half of the relative abundance of the combined communities, but were not detected in the feed above the selected threshold. The feed and full guts shared 103 ASVs which contributed to 27.6% of the overall relative abundance between samples. Separately, the full gut and feed harboured 242 and 248 ASVs not shared with the other sample types, contributing to 4.6% and 14.3% of the relative abundance, respectively.

### Spatial distribution of gut symbionts

**Figure 3.**
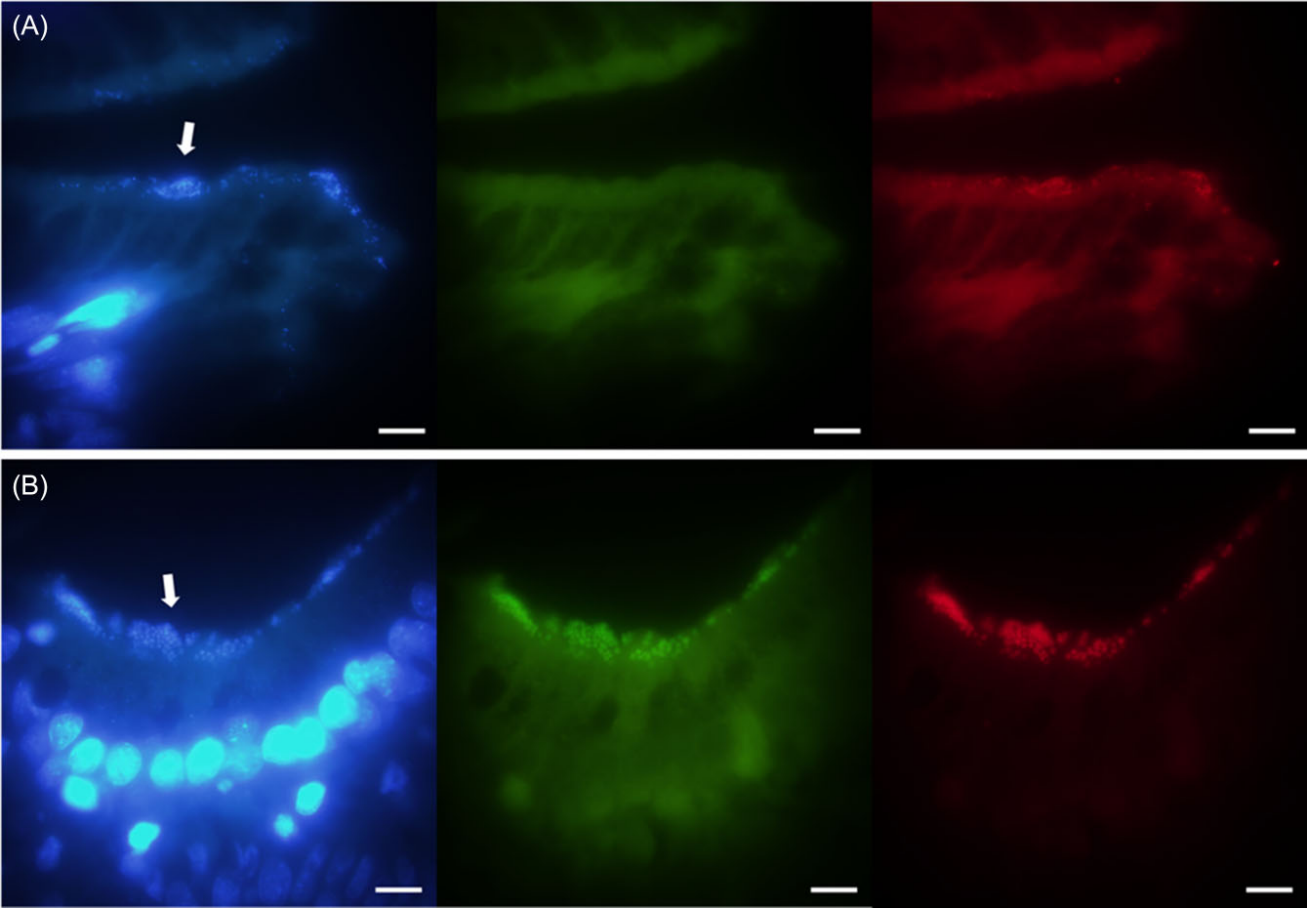
FISH images of hind-gut sections with Mycoplasma-specific probes (A) and Ruminococcaceae-specific probes (B). Left: DAPI stain; middle: universal bacterial probes labelled with Alexa488 (green); right: taxon-specific probes labelled with Cy3 (red). Arrow pointing toward bacterial cells lining the epithelium. The sections shown were taken from fish at time point T2. Bar: 10 µm.

Microscopic observation of the gut microbiota at all time points using 16S rRNA FISH showed colonization of the gut epithelia by *Mycoplasma* sp. and the unclassified *Ruminococcaceae*. The *Brevinema* sp. was not detected using the selected probes, possibly due to site inaccessibility of the targeted 16S rRNA region or a lack of adhesion of the bacteria to the gut epithelium. The *Mycoplasma* sp. appeared slightly elongated with a length of ∼0.6 µm and was distributed as single cells or in clusters of up to 8 µm thickness on the outer layer of the intestinal mucosa (Fig. 3A). However, the epithelium was not covered uniformly with *Mycoplasma* with many areas being void of bacteria. The unclassified *Ruminococcaceae* was spherical-shaped and ∼1 µm in diameter (Fig. 3B). It formed dense clusters up to 10 µm thickness and was found predominantly in fish with empty guts. 16S rRNA FISH confirmed that *Mycoplasma* and the unclassified *Ruminococcaceae* were the dominant bacteria in the gut of the sampled Arctic char, with few other bacterial morphotypes detected with the universal bacterial probes.

### Phylogeny of the dominant gut symbionts and comparison to wild Arctic char

To perform phylogenetics and understand the putative function of the three dominant gut symbionts, DNA samples enriched in *Mycoplasma* sp., *Brevinema* sp., and *Ruminococcaceae* were selected for metagenomic analysis. Assembly and MAG binning of the metagenomic datasets led to the recovery of three medium- to high-quality MAGs [defined according to Bowers et al. (2017)] for the *Mycoplasma* sp., *Brevinema* sp., and unclassified *Ruminococcaceae*, designated AC_MYC01, AC_BRV01, and AC_RUM01, respectively (Table 1).

**Table 1.** Genome characteristics of MAGs AC_MYC01, AC_BRV01, and AC_RUM01.

Genome features and KO groups	AC_MYC01	AC_BRV01	AC_RUM01
Closest cultivated relative [16S rRNA gene similarity (%)]	*Mycoplasma moatsii* (93.77)	*Brevinema andersonii* (90.97)	*Paludicola psychrotolerans* (89.81)
Genome size (bp)	852 238	1 499 402	1 299 186
Contigs	153	18	50
N50	10 527	173 726	94 516
GC content (%)	26.2	29.2	27.1
Genome completeness (%)	96.54	92.13	85.12
Contamination (%)	0.86	0	0
Average genome coverage	15.5	120.9	64.1
ORFs	874	1 397	1 262
tRNA genes	24	30	31
rRNA genes	5S/16S*	5S/16S/23S	5S/16S*
COGs	522	896	832
KOs	430	723	730
CAZy families	5	31	25
GenBank accession number	JAGUQV000000000	JAGUQW000000000	JAGUQX000000000

* indicate genes reconstructed manually from the metagenome.

**Figure 4.**
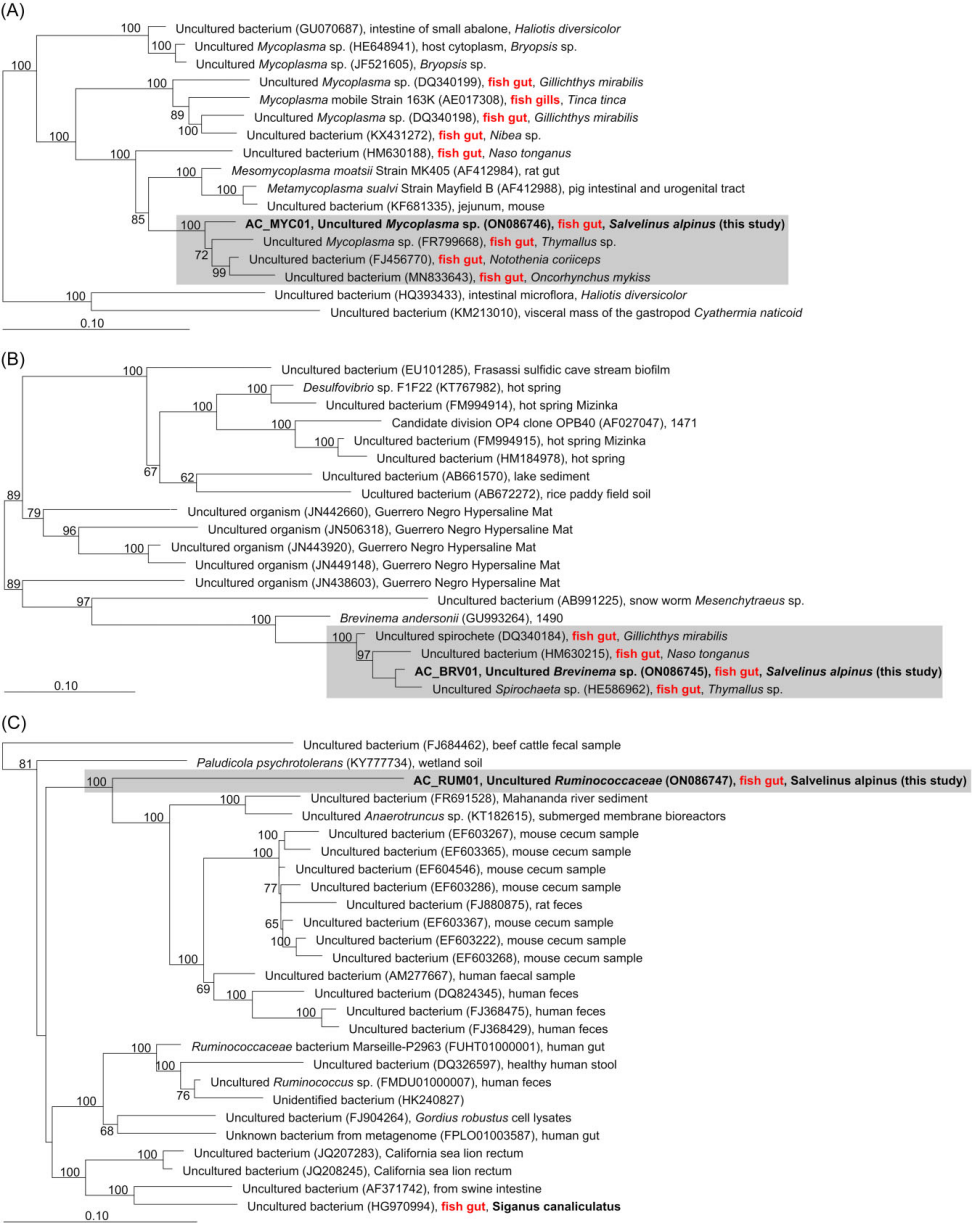
Maximum-likelihood phylogenetic trees for AC_MYC01 (A), AC_BRV01 (B), and AC_RUM01 (C) with related bacteria based on near full-length 16S rRNA gene sequences. Bootstrap values (>50%) are given as percentages at the branching points and are based on 100 resamplings. Related sequences of bacteria retrieved from fish are marked in bold. Bars show 0.10 substitutions per nucleotide position.

The closest cultivated relatives to the AC_MYC01, AC_BRV01, and AC_RUM01 based on near full-length 16S rRNA gene sequence similarity, were *Mycoplasma moatsii* (93.45% sequence identity), *Brevinema andersonii* (90.97%), and *Paludicola psychrotolerans* (89.81%), respectively. Comparison of average AAI to members of the respective or closely related genera showed less than 56% to each of the MAGs (Table S1, Supporting Information), below the suggested threshold of 65% for species of shared genera (Konstantinidis and Tiedje 2007), indicating that they likely fall within novel genera. However, for this study, they will continue to be named according to the taxonomic classification of their respective ASVs. Alignment against the nonredundant Silva 138 SSU database and phylogenetic analysis placed the AC_MYC01 and AC_BRV01 separately into clades with three other uncultivated bacteria previously detected in the gut of fish species and within the order *Mycoplasmatales* and *Brevinematales*, respectively (Fig. 4A and B). AC_RUM01 occupied a separate branch to several uncultured bacteria in the order *Oscillospirales* previously detected in human, animal and environmental samples (Fig. 4C).

To determine if the dominant bacteria detected in farmed Arctic char might be obligate symbionts essential for animal health or gut function, a comparison was made to the gut microbiota of 35 wild juvenile Arctic char. This showed that only the ASV corresponding to the *Ruminococcaceae* bacterium was shared (100% sequence similarity) between the wild and farmed fish (Fig. S2, Supporting Information). In total, 29 of the wild fish harboured this specific *Ruminococcaceae* which contributed to an average relative abundance of 49.4% of the gut microbiota. The second and third most abundant ASVs belonged to the genus *Deefgea* and *Propionibacterium* with 7.6% and 3.8% of the average relative abundance, respectively. Neither *Mycoplasma* nor *Brevinema* were detected in the wild fish gut microbiota.

### Functional attributes of the dominant gut symbionts

An overview of the genome characteristics of AC_MYC01, AC_BRV01, and AC_RUM01 are shown in Table 1. AC_MYC01 had the smallest genome size with 0.85 Mbp, followed by AC_RUM01 with 1.30 Mbp and AC_BRV01 with 1.50 Mbp. This corresponded with the number of detected ORFs, being 874, 1262, and 1397 for AC_MYC01, AC_RUM01, and AC_BRV01, respectively. The GC content ranged from 26.2% for AC_MYC01 to 29.2% for AC_BRV01.

**Figure 5.**
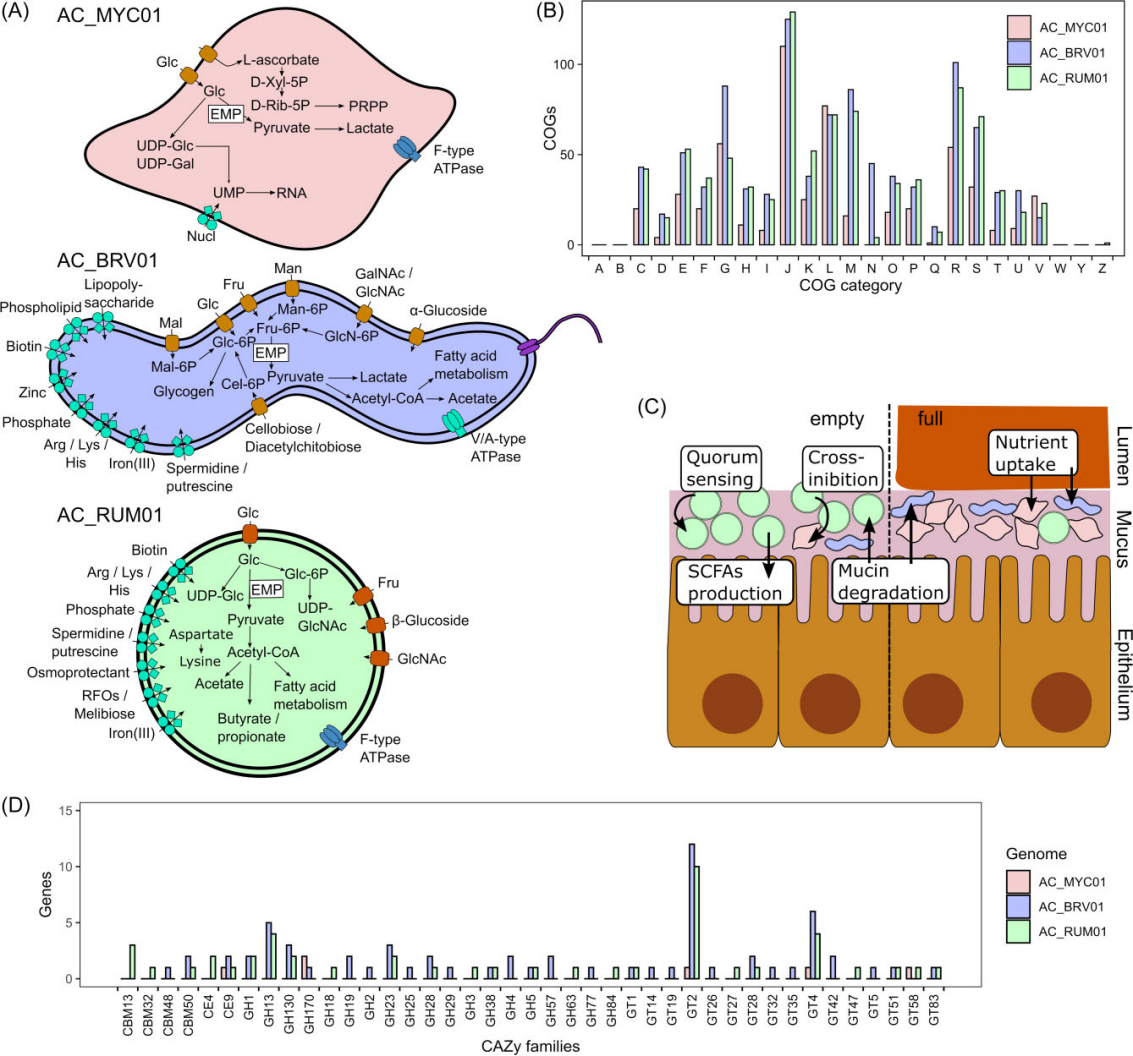
Overview of the functional properties for the MAGs AC_MYC01, AC_BRV01, and AC_RUM01. (A) Hypothesized metabolic pathways reconstructed for the MAGs; (B) number of COGs per COG category for each MAG; COG functional categories: A, RNA processing and modification; B, chromatin structure and dynamics; C, energy production and conversion; D, cell cycle control, cell division, and chromosome partitioning; E, amino acid transport and metabolism; F, nucleotide transport and metabolism; G, carbohydrate transport and metabolism; H, coenzyme transport and metabolism; I, lipid transport and metabolism; J, translation, ribosomal structure, and biogenesis; K, transcription; L, replication, recombination and repair; M, cell wall/membrane/envelop biogenesis; N, cell motility; O, post-translational modification, protein turnover, chaperones; P, inorganic ion transport and metabolism; Q, secondary metabolite biosynthesis, transport, and catabolism; R, general function prediction; S, function unknown; T, signal transduction mechanism; U, intracellular trafficking, secretion, and vesicular transport; V, defence mechanism; Y, nuclear structure; and Z, cytoskeleton. (C) Overview of the putative functions and interaction of the gut microbiome in empty and full states; (D) dbCAN analysis comparing the functional CAZy family classifications in the MAGs.

The genomes of the three dominant bacteria differed in metabolic potential and cellular function with AC_MYC01 having, for instance, comparatively fewer COGs in categories “Energy production and conversion” (COG category C), “Amino acid transport and metabolism” (E) and “Cell wall/membrane/envelop biogenesis” (M), AC_BRV01 having more COGs in category “Cell motility” (N) and AC_RUM01 having fewer COGs in category “Carbohydrate transport and metabolism” (G) compared to their genome sizes (Fig. 5B).

Hypothesized metabolic pathways of the three bacteria are presented in Fig. 5(A) with a complete list of KOs (KEGG Orthology groups) in Table S2 (Supporting Information). In AC_MYC01 the central energy production and carbohydrate metabolism was glycolysis via the Embden–Meyerhof–Parnas (EMP) pathway. It further contained phosphotransferase systems (PTS) for glucose, fructose, and l-ascorbate transport, the former two likely being degraded by the EMP pathway to pyruvate. Pyruvate could be further metabolized to lactate by lactate dehydrogenase, but genes were lacking to metabolize pyruvate to acetyl-CoA. l-ascorbate could be degraded to d-xylulose-5P, an intermediate in the pentose phosphate pathway, with the UlaG enzyme substituted with a lactonase Ms0025 as described for *Mycoplasma synoviae* (Korczynska et al. 2014). Similar to other *Mycoplasma* spp., AC_MYC01 did not have complete pathways for amino acid biosynthesis (Himmelreich et al. 1996, Arraes et al. 2007, Santos et al. 2011). However, it contained a complete pyrimidine ribonucleotide biosynthesis pathway and both nucleotide sugar biosynthesis pathways for glucose to UDP-glucose and galactose to UDP-galactose were present, as well as the interconversion of UDP-glucose to UDP-galactose through UDP-glucose 4-epimerase. The genome further contained all genes involved in F-type ATPase which could be involved in gliding motility on host cells as previously described for other *Mycoplasma* (Tulum et al. 2020). AC_MYC01 only contained genes coding for five carbohydrate active enzyme (CAZy) families, namely Carbohydrate Esterase Family 9 (CE9), Glycoside Hydrolase Family 170 (GH170), and Glycosyl Transferase Family 2, 4, and 58 (GT2, GT4, and GT58) (Fig. 5D).

In AC_BRV01, glycolysis was also the central energy and carbohydrate metabolism. It contained various sugar transporters, including PTS for glucose, maltose, fructose, mannose, cellobiose or diacetylchitobiose, alpha-glucoside, *N*-acetylgalactosamine, and *N*-acetylglucosamine. The latter two substrates being present in large amounts in mucin (Liu et al. 2021) and hence could point towards mucin utilization and degradation. This is further highlighted by the presence of several genes coding for glycoside hydrolase, including GH2 β-galactosidases that can cleave linkages of Gal-β1,3-GalNAc and GH29 fucosidases that can cleave fucose linked to mucin *O*-glycans (Raba and Luis 2023). AC_BRV01 also contained genes for converting mannose and *N*-acetylglucosamine to d-fructose-6-phosphate via mannose-6-phosphate isomerase and *N*-acetylglucosamine-6-phosphate deacetylase, respectively, as well as for maltose and cellobiose to d-glucose-6-phosphate via maltose-6′-phosphate and 6-phospho-beta-glucosidase, respectively. Hence, AC_BRV01 could use several substrates for energy production. AC_BRV01 could further convert pyruvate to acetyl-CoA and to acetate via phosphate acetyltransferase and acetate kinase. Similar to the *Mycoplasma* sp., AC_BRV01 did not have any complete known pathways for amino acid biosynthesis, instead containing an ABC transporter for arginine, lysine, and histidine. It also contained most genes in the initiation pathway of fatty acid biosynthesis and ABC transporters for phospholipids and lipopolysaccharides. AC_BRV01 had several genes required for flagella assembly and chemotaxis, which could confer it mobility.

The unclassified *Ruminococcaceae* AC_RUM01 contained full pathways for pyruvate oxidation, phosphoribosyl diphosphate (PRPP) biosynthesis, nucleotide sugar biosynthesis, and UDP-*N*-acetyl-d-glucosamine biosynthesis. Acetyl-CoA from pyruvate oxidation could be further converted to acetate, via the phosphate acetyltransferase-acetate kinase pathway. AC_RUM01 contained all genes involved in fatty acid biosynthesis, as well as multiple genes in the porA pathway and pyruvate to acetate-formate gene clusters (Fig. S3, Supporting Information) involved in the production of short-chain fatty acids (Amador-Noguez et al. 2010, Guo et al. 2019). The genome further contained all genes for lysine biosynthesis and the Shikimate pathway, producing chorismite, a precursor for aromatic compounds. AC_RUM01 had genes coding for several ABC transporters and PTS including those for the amino acids arginine, lysine, and histidine. It also contained a PTS for *N*-acetylglucosamine. and a gene coding for Glycoside Hydrolase Family 84 (GH84) β-*N*-acetylglucosaminidases potentially cleaving GlcNAc, indicating the utilization of by-products from previously degraded mucin (Raba and Luis 2023). Compared to the other two genomes, AC_RUM01 contained secondary metabolite biosynthetic gene clusters. These were predicted to produce a cyclic-lactone-autoinducer, a ranthipeptide, and an unspecified ribosomally synthesized and post-translationally modified peptide product (RIPP), involved in quorum sensing and cross-inhibition (Sturme et al. 2002, Chen et al. 2020) (Fig. S4, Supporting Information).

## Discussion

Using a longitudinal approach, it is shown that the gut microbiota of farmed Arctic char changes within the first weeks post hatch, similar to previous reports on the succession of the gut microbiota in fish (Bledsoe et al. 2016, Keating et al. 2021). Due to the relatively short period of investigation, it was not possible to conclude when or if the gut microbial community had reached a stable state. However, *Mycoplasma* remained the dominant taxon throughout the study period, followed by *Brevinema* and *Ruminococcaceae*, the latter two becoming more abundant as age progressed. This community profile differed, but also showed similarities, to those of previously described wild and farmed Arctic char, which can vary widely depending on geographic location, habitat and diet (Nyman et al. 2017, Hamilton et al. 2019, Element et al. 2020, 2021). Nyman et al. (2017) analysed the gut microbiota of on-growing Arctic char fed experimental and control diets, showing that the dominant taxa were *Photobacterium* and *Leuconostocaceae*, with a mean relative abundance of 14.2% and 13.6%, respectively. *Mycoplasma, Brevinema*, and *Ruminococcaceae* were absent from the gut community. In contrast, Element et al. (2020) also detected *Mycoplasma* and *Brevinema* as two dominant taxa in the gut of wild Arctic char, with average relative abundances varying, depending on the habitat of the fish. Phylogenetic analysis and a comparison to wild fish from Icelandic waters, provide further evidence that the three dominant gut taxa in this study are fish-associated bacteria, while highlighting likely species or strain-level differences between hosts. The *Ruminococcaceae* strain, in particular, could have coevolved with wild Arctic char in Iceland and confer certain benefits to its host, as previously suggested for other fish symbionts (Kim et al. 2021, Rasmussen et al. 2023). Detection of the three dominant strains on the fish skin in high relative abundances could be explained by a transfer of the strains from faecal matter in the tank water to the skin and their affinity to adhered to a surface as biofilms. However, a low abundance of the taxa in the tank water and higher abundance on the skin could also point towards the bacteria having more than one niche or modes of transmission.

The large inter-individual variability of the gut microbiota detected in the present study has also been described for gut microbiota of other fish species (Gatesoupe et al. 2016, Knobloch et al. 2021) and likely reflects the presence of only few stable taxa and the detection of otherwise allochthonous, or transient, bacteria. A comparison to the feed samples underpins this hypothesis, as apart from the three dominant taxa, only one bacterium, a *Rhodococcus*, was shared between full and empty guts while not being detected in the feed samples. A study by Karlsen et al. (2022) showed that the autochthonous gut microbiota of Atlantic salmon was much less diverse if excluding feed-associated bacteria. In their study, both *Mycoplasma* and *Ruminococcaceae* were the dominant digesta-specific taxa in the gut. However, it appeared that a dominance of *Mycoplasma* precluded a high relative abundance of *Ruminococcaceae* and vice versa. This supports our findings that *Mycoplasma* and *Ruminococcaceae* alternate in high relative abundance due to environmental changes in the gut environment. In the present study, the dominance of *Ruminococcaceae* was clearly linked to an empty mid- and hind-gut and points toward a shift in community structure depending on nutrient availability. In their study on wild Arctic char, Element et al. (2020) found that *Mycoplasma* and *Brevinema* both had a lower relative abundance in overwintering fish compared to those in the other seasons. This suggests that other taxa, possibly with similar functions to the unclassified *Ruminococcaceae*, take over during such period of possible prolonged fasting in the wild.

16S rRNA FISH analysis showed that both *Mycoplasma* and *Ruminococcaceae* build thick clusters of cells in the mucus layer on the intestinal epithelium, which could facilitate nutrient exchange, but also enable rapid removal once the mucus is shed (Ringø et al. 2007). A similar spatial occurrence of bacterial cells on the epithelial surface was observed in the distal gut of juvenile rainbow trout in which *Mycoplasma* was also the dominant taxon (Rasmussen et al. 2021). In addition, a study by Cheaib et al. (2021), employing 16S rRNA FISH, showed that *Mycoplasma* also inhabited areas further up the digestive tract in Atlantic salmon, including the stomach lining and pyloric caecum. Whereas not studied in the present work, these sites could be potential reservoirs leading to the rapid recolonization of *Mycoplasma* in the mid- and hind-gut when digesta passes through the intestine.

Genome analysis of the three dominant gut symbionts showed that the gut microbiome of farmed Arctic char could provide several benefits to its host. Similar to other *Mycoplasma*, the genome of AC_MYC01 was small with few specialized metabolic pathways. This reduction of genes has previously been associated with a high adaptation to a host-associated lifestyle (Cheaib et al. 2021), thus not necessitating endogenous biosynthesis in an environment characterized by high nutrient availability. The lack of pathways involved in the biosynthesis of amino acids and vitamins, both previously detected in salmonid-related *Mycoplasma* (Rasmussen et al. 2021, 2023), further suggests that these are not the main reasons for the symbiotic relationship between *Mycoplasma* and salmonids. Instead, its small genome size, adapted to the host gut environment, paired with the ability to rapidly recolonize the gut after less favorable conditions, could be the main feature enabling this symbiosis.

The *Brevinema* sp. had similar gene functions to *Mycoplasma*, albeit a higher number of sugar and other nutrient transporters. This along with potential mobility could confer it a spatial niche apart from *Mycoplasma* in the mucus lining, while enabling cross-feeding and efficient scavenging for available nutrients. *Brevinema* is often detected as a salmonid-associated gut symbiont (Brown et al. 2019, Gupta et al. 2019, Li et al. 2021), but its functional attributes have so far remained elusive. The draft genome of AC_BRV01 highlights the potential role of *Brevinema* as a well-adapted nutrient scavenger and cohabitant of the salmonid gut along with *Mycoplasma*.

Members of the class *Clostridia*, including *Ruminococcaceae*, are often involved in SCFA production in animal intestines (Barcenilla et al. 2000, Zhang et al. 2018, Lan et al. 2023). The genome of AC_RUM01 contained genes involved in SCFA production, which could be beneficial to the host (Li et al. 2022). Although the guts were characterized as empty, remnants of the digesta needed for SCFA production were likely still present due to continuous feeding, and hence rapid turnover of nutrients in the gut. In addition, its genome contained genes involved in mucin degradation and utilization of by-products from previously degraded mucin. This could be an additional energy source and confer it a growth advantage when other nutrients become limited once the digesta has left the mid- and hind-gut. Interspecies cross-feeding of gut microorganisms has previously been described in other animals (Solden et al. 2018, Luo et al. 2021) and could aid in creating a stable microbial community composition, making it less susceptible to invasion or perturbation from external sources (Culp and Goodman 2023). Furthermore, degradation and feeding on mucin is a factor for maintaining a protective barrier and contributing to intestinal homeostasis, thereby contributing to host health (Paone and Cani 2020). Further, AC_RUM01 contained full pathways for the biosynthesis of amino acids, possibly enabling its rapid growth even in nutrient limited conditions. The takeover of the gut microbiome by *Ruminococcaceae* when the gut is temporarily empty could be coordinated be the production of secondary metabolites enabling rapid proliferation and suppression of other species when conditions are suitable (Uhlig and Hyland 2022). Such clear alternation in relative abundance between dominant gut symbionts has not yet been shown for fish and points toward a strategy that could prevent the colonization of harmful bacteria in a fluctuating and dynamic environment.

## Conclusion

This study provides an in-depth overview of the gut microbiota in farmed Arctic char and the functional attributes of its dominant resident symbionts. These insights are also relevant for other salmonid species due to the frequent presence of these bacterial taxa among salmonid gut microbiota. We show that there is a clear alternation in the relative abundance of the main symbionts depending on passage of the digesta with different functional characteristics possibly adapted to either a state of high or low nutrient availability. This presents a previously undiscovered strategy to maintain a high bacterial abundance in the gut during fluctuating environmental conditions and thereby prevent colonization of microorganisms that could be harmful to the host. The presence of the same sequence type of an unclassified *Ruminococcaceae* in both wild and farmed Arctic char further raises the questions of how essential this bacterium is for host health or disease resistance. Further research is also needed to better understand the interactions between the cohabitating *Mycoplasma* and *Brevinema*, as well as their impact on host health and productivity. Targeted growth studies and pathogen challenge experiments between fish with and without each symbiont will provide further insights into these questions, as well as the into the potential of targeted gut microbiome modulation for improved aquaculture performance of salmonid species.

## Supplementary Material

xtae011_Supplemental_Files

## Data Availability

Raw 16S rRNA gene amplicon data of farmed and wild Arctic char is deposited in the NCBI SRA under BioProject PRJNA720530, BioSamples SAMN35552807–SAMN35552919, and under BioProject PRJNA791800, BioSamples SAMN24345287–SAMN24345273 and SAMN24345352–SAMN24345338. Raw metagenomic sequences of three gut samples used for MAG binning are deposited in the NCBI SRA under BioProject PRJNA720530, accession SRR24775306–SRR24775308. Near full-length 16S rRNA gene sequences of AC_MYC01, AC_BRV01, and AC_RUM01 are deposited under NCBI GenBank accessions ON086746.1, ON086745.1 and ON086747.1, respectively. Draft genome assemblies of AC_MYC01, AC_BRV01, and AC_RUM01 are deposited at DDBJ/ENA/GenBank under accessions JAGUQV000000000, JAGUQW000000000, and JAGUQX000000000, respectively. The versions described in this paper are versions JAGUQV010000000.1, JAGUQW010000000.1, and JAGUQX020000000.1.
