# Supplementary material for: The gut microbiome of farmed Arctic char (Salvelinus alpinus) is shaped by feeding stage and nutrient presence
Source: FEMS Microbes. 2024 Apr 23;5:xtae011. doi: 10.1093/femsmc/xtae011 (PMC11092275; doi:10.1093/femsmc/xtae011)
Supplement: xtae011_Supplemental_Files [file xtae011_supplemental_files.zip › FEMSMC-2023-047.R1 one sentence summary.docx]

The gut microbiome of farmed Arctic char (Salvelinus alpinus) is dominated by three bacterial taxa which alternate in relative abundance based on feed availability.
