# Supplementary material for: The gut microbiome of farmed Arctic char (Salvelinus alpinus) is shaped by feeding stage and nutrient presence
Source: FEMS Microbes. 2024 Apr 23;5:xtae011. doi: 10.1093/femsmc/xtae011 (PMC11092275; doi:10.1093/femsmc/xtae011)
Supplement: xtae011_Supplemental_Files [file xtae011_supplemental_files.zip › Supplemental_Figures.docx]

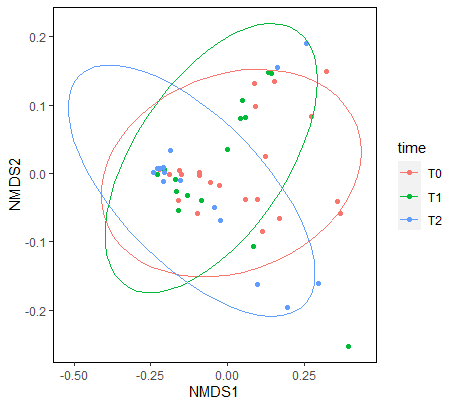


**Supplemental Figure S1**: Non-metric Multi-dimensional Scaling (NMDS) plot of weighted UniFrac distances between gut microbial communities (stress = 0.10).


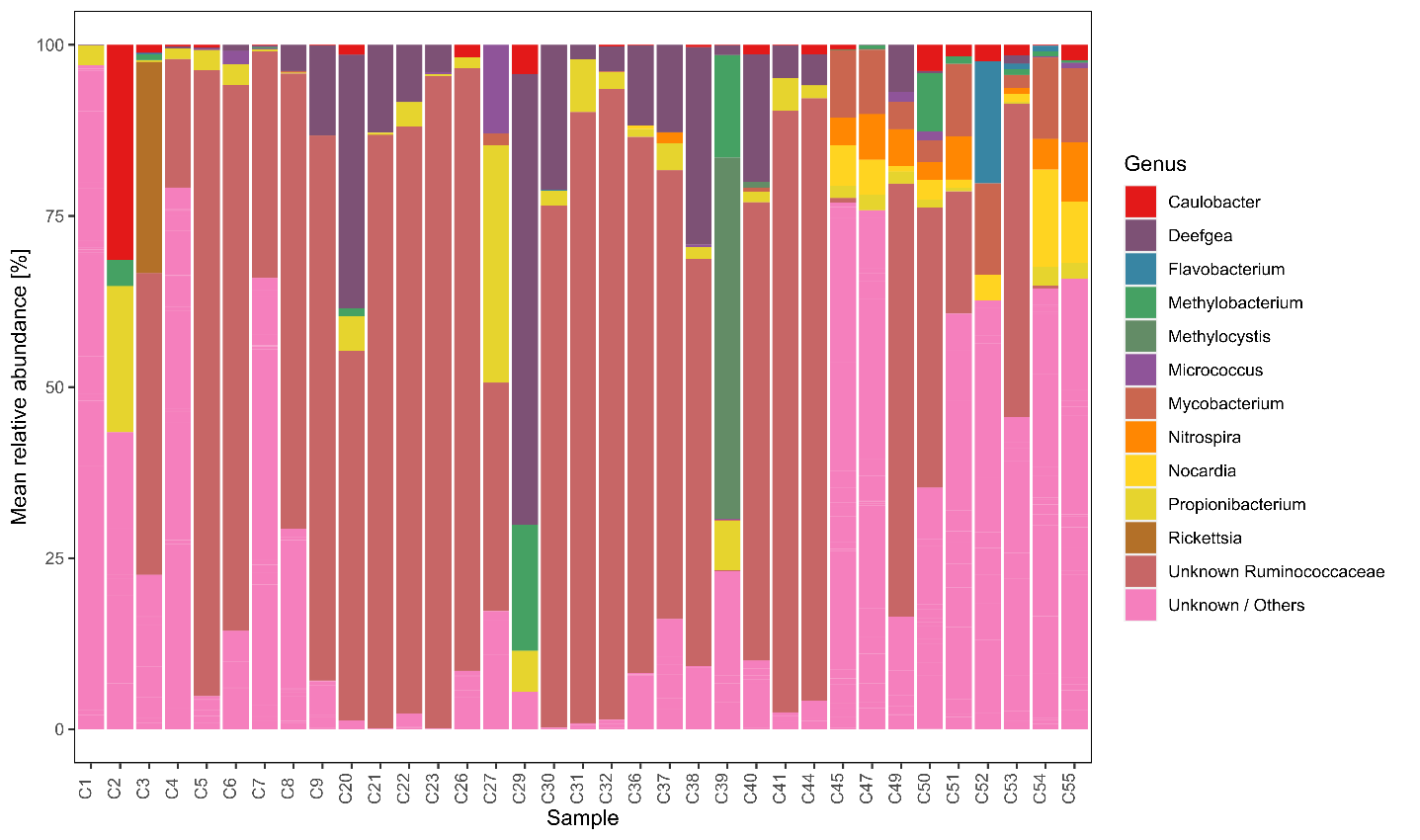


**Supplemental Figure S2:** Microbial community composition of 35 wild Arctic char collected from fresh water springs in Iceland. Genera with less than 0.5% average relative abundance across all samples and unassigned taxa are summarised under “Unknown / Others”.


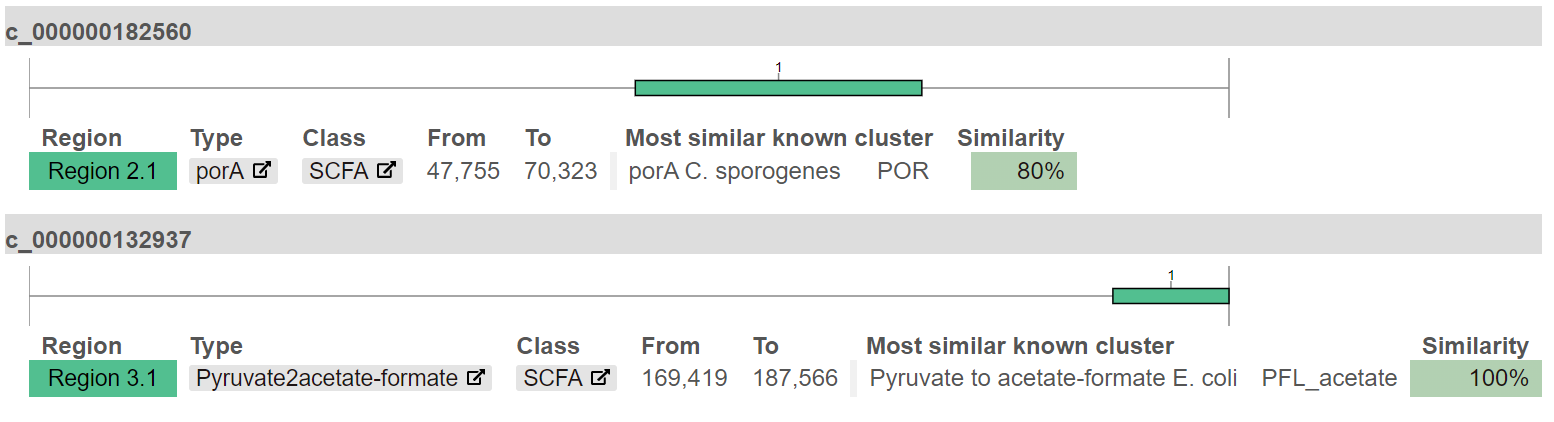


**Supplemental Figure S3**: gutSMASH results of AC_RUM01.


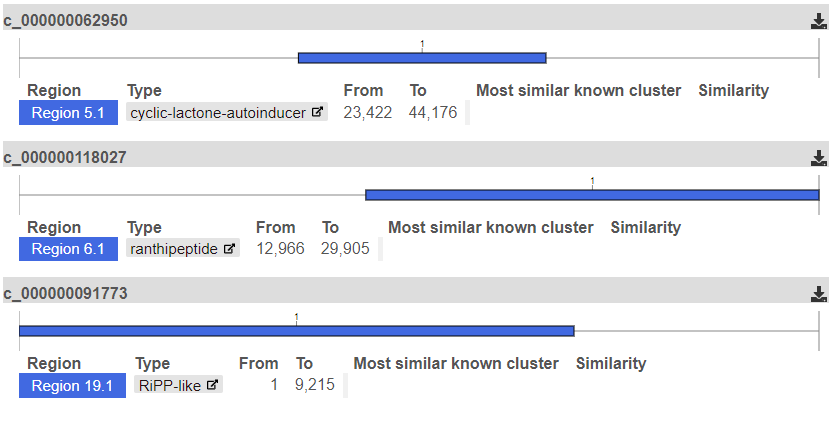


**Supplemental Figure S4**: antiSMASH results of AC_RUM01.
