# Supplementary material for: The gut microbiome of farmed Arctic char (Salvelinus alpinus) is shaped by feeding stage and nutrient presence
Source: FEMS Microbes. 2024 Apr 23;5:xtae011. doi: 10.1093/femsmc/xtae011 (PMC11092275; doi:10.1093/femsmc/xtae011)
Supplement: xtae011_Supplemental_Files [file xtae011_supplemental_files.zip › FEMSMC-2023-047.r1 supplementary data - reviews and author response.docx]

**Reviews of manuscript FEMSMC-2023-047**

**Reviewer 1: Andri Eirikur, University of Copenhagen**

**Recommendation: Minor Revision**

This very interesting study reports on the gut microbiome of juvenile Arctic char and its main symbionts. The paper is well written, clear, and well structured. The inclusion of environmental water and feed controls is commendable. The additional comparison of full vs. empty guts along with the includsion of wild Arctic char adds rigor to the study. Overall, the paper will be a valuable addition to the rather scarce but growing pool of fish microbiome studies. However, several aspects of the paper require further clarifications and justifications before being considered for publication.

It should be noted that I have not thoroughly reviewed the section on Fluorescence in situ hybridization, as I am not experienced enough with the FISH method to provide expert opinion (although I have included one minor comment on that image).

Line 86-88: “[...] average weight of 2.2g to 9.8g [...]”. Please clarify what this range represents. If it is the average of the T0 and the T2, include the average of T1 rather than presenting the averages in a range. Furthermore, was there a reason for why these three time points were chosen? If so, clarify.

Lines 98-99:

i) The authors note in the results “the presence of Brevinema and unclassified Ruminococcaceae appeared to increase over time”. I realise this is a difficult thing to address, however, I wonder if the increase in these genera across the time points, particularly Ruminococcaceae, is linked to feeding or empty/whole gut, rather than age. Was the time between feeding and sampling synchronized for the sample points? Was the empty/full state of the gut recorded for all of the fish?

i) In the same sentence “[…] with a higher percentage of fish containing both species […]”, please include the percentage difference.

Lines 121-153 (Pertaining to results section 2.2 and figure 2). This is overall a nice analysis and great and important to see feed controls included. However, some clarifications are needed on the following:

i) Line 131-133: What variables were used in the PERMANOVA presented “(PERMANOVA, F(3,31)=26.736,p=0.001)”? Since a significant difference is mentioned between both full and empty guts, and gut and feed samples the significance value of the PERMANOVA is difficult to interpret (although the mentioned differences are clear in the ordination space).

ii) For clarity, be consistent in how you report on the three gut groups in the text and figures. For example, significant differences between full and empty guts are reported based on the PERMANOVA mentioned above, while the figure (2E) also has a “partial” group, which groups with the full guts, indicating that full and partial have been grouped together. Similarly is the “full” group in the venn diagram (2C) a merger of “full” and “partial”? If so, that should be clarified.

iii) Assuming that the percentages in the Venn diagram (Figure 2C) are relative abundances across the dataset (guts plus feed), I am not sure they give much insight into differences between samples. Take for example, that the four dominant ASVs shared between full and empty guts represent 51.2% of the total relative abundance. This percentage is affected by the absence of those ASVs in the feed. It would be more meaningful to see the relative abundance of the four ASVs in the full gut and empty gut groups separately. This also applies to the interesting fraction of ASVs shared only between feed and full gut. It would be very interesting to know the fraction of feed ASVs in the full guts since it’s a very often overlooked factor.

Line 169 (Figure 3): Nice images, I wonder if the far right figure 3A the quality or brightness can be increased as the mycoplasma signals appear quite faint.

lines 175-207: Nice section, really nice to include some wild chars. I wonder if doing phylogeny on the whole draft genomes assembled, instead of the partial 16S sequence, was considered. There would then be possibility of, among other things, calculating the average nucleotide identity (ANI), compared to the close relatives to better place them as potentially novel genera.

lines 220-224 and figure 5B requie clarifications on the following:

i) […] AC_MYC01 having comparatively fewer COGs […] AC_BRV01 having more COGs […] AC_RUM01 having fewer COGs[…] compared to their genome sizes. It is unclear what is being compared here and in what context. As it seems on figure 5B, mycoplasma has comparatively fewer COGs in other than just the three categories mentioned in text.

ii) The COG categories mentioned in text don´t reference to the figure (i.e. it is not clear that ‘Energy production and conversion’ corresponds to C on the figure).

iii) There is a mismatch between A)-D) numbering in the figure caption and the figure itself.

Lines 393-397: Were the char in the same tank or distributed in different tanks for each time point? Is there a chance the difference between time points be a tank effect? Please clarify.

In lines 122-125: It is noted that 24 additional samples were acquired from T2 for the empty/full gut comparison. A text regarding those samples is missing in the methods.

Lines 448-452: Was there any host-DNA filtering done for the metagenomics reads prior to assembly?

Lines 452-453: Please be more specific about the binning approach. Was it manual binning or automatic binning or combination? (and if automatic binning was applied include a reference).

The notion of the potential dynamics of the Mycoplasma and the Ruminococcaceae in gut environmental changes is highly interesting. A “rapid recolonization” of Mycoplasma is claimed several times throughout the manuscript. Is there evidence supporting that notion apart from the differences in abundance between empty and full guts? This claim needs further discussion and clarification. Since the Mycoplasma is present in all age group samples, I wonder, instead of rapid recolonization, if Mycoplasma is never really absent but is constantly present in a low absolute stable abundance while the Ruminococcaceae grows in absolute abundance in the more empty gut.

**Reviewer 2**

**Recommendation: Major Revision**

The paper aims to identify the gut microbial community of farmed Arctic charr and describe its functionality using amplicon 16S sequencing, fluorescence in situ hybridization and metagenomic analysis. The work focuses on three dominant microbial taxa that are abundant in the gut at juvenile stages of Arctic charr. The study gives novel insights into the functional role of dominant taxa in farmed Arctic charr, which is a good start to better understand the role of those core abundant taxa.

Overall, the study is clear and well-written, with conclusions that are mostly substantiated by the results. Some points still need clarification:

Like 79-80: at which stage is the fish from 104-160 days post hatch? This indicates approximately 3 – 4 months old fish. I would assume this means it is juvenile most likely. So it would be best to report this clearly. The wording first weeks of life would indicate first feeding or larvae/post larvae stages. It is not clear if this would be the case.

Figure 5B – please explain which of the bars fall into the empty and the full gut scenarios. This is not very clear.

Line 397-405: When sampling for the gut microbiota, as the authors also observe, it is quite important to understand whether the community comes from digesta or mucosa, and whether fish were fed or not in this case, because this can largely affect the taxa present and their abundance. Can you provide details on whether the mucus, whole gut or digesta was collected? Also it is not always clear, is it the hindgut always that was sampled for the DNA extraction?

Line 448: Usually there is quite some host DNA in the gut samples (depends what you sampled in this case: mucus vs digesta). How did you remove the host DNA from the analysis?

Line 479 – 480: Please provide a more detailed protocol on the hybridization steps. Or at least a reference?

Discussion: Maybe I missed this part (only see being addressed briefly in lines 295-297) but in many studies they report that Mycoplasma is actually also associated with wild salmonids. In the present study, this was not the case when compared to wild fish. Can the authors address this issue, or give suggestions?

Title: To me this was not too clear what this was about from the title. I would propose the authors to come up with a more specific message for their study and title. In the end, it was very much related to whether the fish had a full or partially full gut or not. So it looks like it is very much associated with feeding stage and nutrient presence.

**Editor Decision: Minor Revision**

**Author's Response to Decision Letter for (FEMSMC-2023-047)**

We thank both reviewers for considering our manuscript for revision and for the helpful comments and suggestions on improving the manuscript.

We have addressed all the reviewers’ comments below with line numbers referring to the revised manuscript without track changes.

Please note that we have changed the title, as requested by reviewer 2, to “The gut microbiome of farmed Arctic char (Salvelinus alpinus) is shaped by feeding stage and nutrient presence”

Responses to the reviewers:

Reviewer: 1

Line 86-88: “[...] average weight of 2.2g to 9.8g [...]”. Please clarify what this range represents. If it is the average of the T0 and the T2, include the average of T1 rather than presenting the averages in a range. Furthermore, was there a reason for why these three time points were chosen? If so, clarify.

Response by the authors: We have changed this part to “(…) with an average weight of 2.2 ± 0.2 g at time T0, 4.5 ± 1.1 g at time T1 and 9.8 ± 0.7 g at time T2.” to give the mean weight ± stdev for each time point (Lines 87-88).

The reason for selecting the time points is given in the Material and Methods section as “Juvenile Arctic char (Salvelinus alpinus) were collected from an ongoing feeding trial (24) at 104 dph (days post hatch) (T0, N = 22), 132 dph (T1, N = 15) and 157 dph (T2, N = 15).” (Lines 402 - 403).

Lines 98-99:

i) The authors note in the results “the presence of Brevinema and unclassified Ruminococcaceae appeared to increase over time”. I realise this is a difficult thing to address, however, I wonder if the increase in these genera across the time points, particularly Ruminococcaceae, is linked to feeding or empty/whole gut, rather than age. Was the time between feeding and sampling synchronized for the sample points? Was the empty/full state of the gut recorded for all of the fish?

Response by the authors: We understand your concern, however, the fish were all samples in the same way for each time point and had received the same feeding regime (see Lines 405 - 408). We therefore think that it is unlikely that this played a role in the different relative abundance profiles between time points. However, we did not record the empty/full state for each fish analysed in this section (hence the following section on gut content).

i) In the same sentence “[…] with a higher percentage of fish containing both species […]”, please include the percentage difference.

Response by the authors: We included the percentages and also made the sentence clearer by stating “containing either one of the species” (Lines 98 - 99)

Lines 121-153 (Pertaining to results section 2.2 and figure 2). This is overall a nice analysis and great and important to see feed controls included. However, some clarifications are needed on the following:

i) Line 131-133: What variables were used in the PERMANOVA presented “(PERMANOVA, F(3,31)=26.736,p=0.001)”? Since a significant difference is mentioned between both full and empty guts, and gut and feed samples the significance value of the PERMANOVA is difficult to interpret (although the mentioned differences are clear in the ordination space).

Response by the authors: We have clarified this section by stating that the PERMANOVA was significantly different between the tested groups (“full”, “partial”, “empty”, “feed”):

“(…) significant difference in the microbial community composition within the gut content and feed groups based on Bray-Curtis dissimilarities (PERMANOVA, F(3, 31) = [26.736], p = 0.001), (…)“

And continued by stating the pairwise comparison between the groups:

“(…) with pairwise comparisons showing significant differences between empty and both full and partially filled guts (padj = 0.006 each) and between the feed samples and all three gut groups (padj = 0.006 each), but not between the full and partially filled guts (padj = 1) (Figure 2E).” (Lines 130 - 134).

We also added the following sentence to the methods section: “Pairwise multilevel comparison was conducted with the R package pairwiseAdonis version 0.4.1 (https://github.com/pmartinezarbizu/pairwiseAdonis).“ (Lines 460 - 461)

ii) For clarity, be consistent in how you report on the three gut groups in the text and figures. For example, significant differences between full and empty guts are reported based on the PERMANOVA mentioned above, while the figure (2E) also has a “partial” group, which groups with the full guts, indicating that full and partial have been grouped together. Similarly is the “full” group in the venn diagram (2C) a merger of “full” and “partial”? If so, that should be clarified.

Response by the authors: We have addressed some of the concerns in the previous answer and have additionally added the following statement to the Figure 2C legend “(partially filled gut group excluded)”, to indicate that the “full” and “partial” group were not merged (Line 150).

iii) Assuming that the percentages in the Venn diagram (Figure 2C) are relative abundances across the dataset (guts plus feed), I am not sure they give much insight into differences between samples. Take for example, that the four dominant ASVs shared between full and empty guts represent 51.2% of the total relative abundance. This percentage is affected by the absence of those ASVs in the feed. It would be more meaningful to see the relative abundance of the four ASVs in the full gut and empty gut groups separately. This also applies to the interesting fraction of ASVs shared only between feed and full gut. It would be very interesting to know the fraction of feed ASVs in the full guts since it’s a very often overlooked factor.

Response by the authors: We agree that the relative abundance giving for the ASVs across all groups is less informative, however, this is the default (and only) output of the ampvis2 package. We argue, that the relative abundance of the three main gut microbes within each gut group is displayed sufficiently on Figure 2B. Although not very informative, we have kept the overall relative abundance values in Figure 2C for now, but could remove them if requested.

Line 169 (Figure 3): Nice images, I wonder if the far right figure 3A the quality or brightness can be increased as the mycoplasma signals appear quite faint.

Response by the authors: Unfortunately, this was the highest contrast possible with the FISH processing program “daime”, as the raw image had quite a lot of background signal.

lines 175-207: Nice section, really nice to include some wild chars. I wonder if doing phylogeny on the whole draft genomes assembled, instead of the partial 16S sequence, was considered. There would then be possibility of, among other things, calculating the average nucleotide identity (ANI), compared to the close relatives to better place them as potentially novel genera.

Response by the authors: We performed AAI on the three MAGs with closely related cultivated strains (Lines 185 - 188) to show their distance to related species. However, for the phylogeny we chose the 16S rRNA gene rather than a whole genome comparison in order to compare the MAGs to uncultivated taxa and their reported habitat or source of isolation, which we thought would be more informative than a more precise taxonomic placement.

lines 220-224 and figure 5B requie clarifications on the following:

i) […] AC_MYC01 having comparatively fewer COGs […] AC_BRV01 having more COGs […] AC_RUM01 having fewer COGs[…] compared to their genome sizes. It is unclear what is being compared here and in what context. As it seems on figure 5B, mycoplasma has comparatively fewer COGs in other than just the three categories mentioned in text.

Response by the authors: We have included the words “for instance” to highlight that this is not a conclusive list of differences but just a highlight of some COG categories that differ in number between the three taxa compared to their genome size:

“(…) AC_MYC01 having, for instance, comparatively fewer COGs in categories (…)” (Lines 222).

ii) The COG categories mentioned in text don´t reference to the figure (i.e. it is not clear that ‘Energy production and conversion’ corresponds to C on the figure).

Response by the authors: Thank you for pointing this out. We have added the COG categories to the Figure 5 legend and have referenced them in the text (Lines 221 - 226).

iii) There is a mismatch between A)-D) numbering in the figure caption and the figure itself.

Response by the authors: We now placed the A)-D) numbering in front of each description.

e.g.: “A) Hypothesised metabolic pathways reconstructed for the MAGs;” (Lines 230 - 241).

Lines 393-397: Were the char in the same tank or distributed in different tanks for each time point? Is there a chance the difference between time points be a tank effect? Please clarify.

Response by the authors: The fish were held in different replicate tanks and fish from each tank were collected at each time point to prevent a tank effect. This is described in the previous publication by Knobloch et al. (Reference 24). However, we agree that is should also be mentioned in this manuscript. We clarify this in lines 403 – 405: “Before the beginning of the feeding trial (T0), one fish was collected from each of the 22 experimental tanks. At T1 and T2, five fish were collected from each of the three replicate control tanks.”

In lines 122-125: It is noted that 24 additional samples were acquired from T2 for the empty/full gut comparison. A text regarding those samples is missing in the methods.

Response by the authors: We added the following text in the methods section: “To compare the gut microbial community in relation to gut content 24 additional fish from time point T2, which had been collected and frozen at -80°C at the end of the experiment, were dissected as mentioned above. The state of digestive content was described as “full” if digesta was present along the mid- and hind-gut section, as “partially full” if digesta was present in parts of the mid- or hind-gut section, and as “empty” if no digesta was observable.” (Lines 420 – 424).

Lines 448-452: Was there any host-DNA filtering done for the metagenomics reads prior to assembly?

Response by the authors: Bacterial DNA was enriched prior to sequencing as stated in lines 443 – 444. Apart from that, no host-DNA filtering was performed. Our consideration was that the coverage would be too low for meaningful assembly of the host genome. Hence, no large contigs (apart from the mitochondrial DNA) were assembled for the host and therefore did not interfere with MAG binning.

Lines 452-453: Please be more specific about the binning approach. Was it manual binning or automatic binning or combination? (and if automatic binning was applied include a reference).

Response by the authors: The binning process was manual. We have added additional information to the methods section (Lines 472 - 479):

“Binning of metagenome-assembled genomes (MAGs) was performed in Anvi’o version 7 (74) following the “Anvi'o User Tutorial for Metagenomic Workflow” (https://merenlab.org/2016/06/22/anvio-tutorial-v2/). In brief, k-mer frequencies were calculated and open reading frames identified in the contigs using the anvi-gen-contigs-database command. HMM profiles were generated using the command anvi-run-hmms and genes annotated with the command anvi-run-ncbi-cogs. Taxonomic annotation was performed with centrifuge (75). Anvi’o profiles were merged and imported into the anvi’o interactive interface. MAGs were then manually binned based on tetranucleotide frequency, taxonomic assignment and coverage.”

The notion of the potential dynamics of the Mycoplasma and the Ruminococcaceae in gut environmental changes is highly interesting. A “rapid recolonization” of Mycoplasma is claimed several times throughout the manuscript. Is there evidence supporting that notion apart from the differences in abundance between empty and full guts? This claim needs further discussion and clarification. Since the Mycoplasma is present in all age group samples, I wonder, instead of rapid recolonization, if Mycoplasma is never really absent but is constantly present in a low absolute stable abundance while the Ruminococcaceae grows in absolute abundance in the more empty gut.

Response by the authors: Although we did not perform a quantitative analysis of the samples processed for FISH, our observation was that the cells stained with the Ruminococcaceae probe had roughly the same abundance in the “empty” guts than Mycoplasma in the “full” guts. If the absolute abundance of Mycoplasma had indeed stayed the same, we would have expected to see much higher concentrations of Ruminococcaceae in the empty guts, which was not the case. We added the word “relative” to the following sentence: “However, it appeared that a dominance of Mycoplasma precluded a high relative abundance of Ruminococcaceae and vice versa” (Line 326), to prevent the impression we are talking about absolute abundances.

Reviewer: 2

Like 79-80: at which stage is the fish from 104-160 days post hatch? This indicates approximately 3 – 4 months old fish. I would assume this means it is juvenile most likely. So it would be best to report this clearly. The wording first weeks of life would indicate first feeding or larvae/post larvae stages. It is not clear if this would be the case.

Response by the authors: We have removed the statement “over the first weeks of life”. The sentence now reads: “The objective of this study was to describe the microbiome of farmed juvenile Arctic char, identifying the stable members of the resident gut microbiota and analysing their putative role in the fish gut microbiome (…)” (line 78).

Figure 5B – please explain which of the bars fall into the empty and the full gut scenarios. This is not very clear.

Response by the authors: In Figure 5B we show the general number of COGs per COG category for each of the three MAGs (colors red, blue, green). This is independent from the empty or full gut scenarios. Plots 5B and 5C have no relation to each other, the former showing putative genome function and the latter a hypothesised function in the gut based on a full and empty gut scenario.

Line 397-405: When sampling for the gut microbiota, as the authors also observe, it is quite important to understand whether the community comes from digesta or mucosa, and whether fish were fed or not in this case, because this can largely affect the taxa present and their abundance. Can you provide details on whether the mucus, whole gut or digesta was collected? Also it is not always clear, is it the hindgut always that was sampled for the DNA extraction?

Response by the authors: We added the sentence: “The fish were not fed 12 h prior to weighing and sample collection.” (line 408).

We also clarified that DNA was extracted from the entire gut (with digesta if present): “DNA from the whole mid- and hind-gut with digesta, if present, was extracted as previously described in Leeper et al. (60).“ (lines 430 - 431

For FISH, we only collected a small section from the hindgut, as we were restricted to the number of samples that we could process and only very small sections of material can be visualised (see lines 412 - 415).

Line 448: Usually there is quite some host DNA in the gut samples (depends what you sampled in this case: mucus vs digesta). How did you remove the host DNA from the analysis?

Response by the authors: We did not deplete host DNA for the 16S rRNA amplicon approach, as we received good results using the selected primer pair and PCR set-up without off-target amplification. For the shotgun sequencing we first used the NEBNext Microbiome DNA Enrichment Kit (New England Biolabs) to remove host reads (see lines 443 - 445). The remaining reads did not interfere with the MAG binning, as host genome coverage was too low.

Line 479 – 480: Please provide a more detailed protocol on the hybridization steps. Or at least a reference?

Response by the authors: We provide a reference in Knobloch et al. (93) (line 507), which we followed exactly apart from the choice of probes and the concentration of formamide in the hybridisation buffer, which are detailed in line 506 - 511.

Discussion: Maybe I missed this part (only see being addressed briefly in lines 295-297) but in many studies they report that Mycoplasma is actually also associated with wild salmonids. In the present study, this was not the case when compared to wild fish. Can the authors address this issue, or give suggestions?

Response by the authors: Unfortunately, we do not have any evidence to reasonably explain why some wild salmonids contain Mycoplasma and others do not. This might be simply due to geographic distribution and genetic variability of populations, or differences in life style / source of feed. We believe this question needs to be addressed in future studies, possibly with controlled experiments with and without Mycoplasma (which would require Mycoplasma in isolation or antibiotic treatment) to further address its functionality in its host. A less probable, but more alarming suggestion would be that Mycoplasma is a species present in aquaculture systems which have been transferred to wild population through farm escapes in open water systems. As Arctic char in Iceland is farmed in closed system, this might not have occurred yet.

Title: To me this was not too clear what this was about from the title. I would propose the authors to come up with a more specific message for their study and title. In the end, it was very much related to whether the fish had a full or partially full gut or not. So it looks like it is very much associated with feeding stage and nutrient presence.

Response by the authors: We agree that the title could be more descriptive and have therefore changed it to:

“The gut microbiome of farmed Arctic char (Salvelinus alpinus) is shaped by feeding stage and nutrient presence”

**Reviews of manuscript FEMSMC-2023-047.r1**

**Reviewer 1: Andri Eirikur, University of Copenhagen**

**Recommendation: Accept**

The authors have adequately addressed the critical comments pertaining to results presentation and interpretation to justify publication. I greatly appreciate the time and effort of the authors invested to respond to the reviwer comments. The revised manuscript looks good, clear and concise. Below I have included two notes that may be useful for improvement.

*Venn diagram (figure 2C) and text pertaining to feed comparison, lines 135-144:*

We are in agreement that the relative abundance across all groups including feed is rather uninformative. Thus, unless you can tease apart for example the relative abundances of feed related ASVs in the “full” gut only (rather than the overall relative abundance), I suggest the Venn diagram and text pertaining to said analysis be moved to the supplementary material. The main results you want to convey, as you note, are sufficiently displayed in Figure 2B and 2E.

*Host DNA filtering for metagenomics:* Even though microbial enrichment was performed, I would always suggest mapping to the host genome (and keeping the unmapped microbial reads) to remove any remaining host fraction to improve downstream assembly and binning performance.

**Reviewer 2**

**Recommendation: Accept**

All my comments were addressed.

**Editor Decision: Accept**
